# *trips4health*: a single-blinded randomised controlled trial incentivising adult public transport use for physical activity gain

**DOI:** 10.1186/s12966-023-01500-7

**Published:** 2023-08-16

**Authors:** Jack. T. Evans, Oliver Stanesby, Leigh Blizzard, Kim Jose, Melanie J. Sharman, Kylie Ball, Stephen Greaves, Andrew J. Palmer, Katie Cooper, Seana L. Gall, Verity J. Cleland

**Affiliations:** 1 Menzies Institute for Medical Research, University of Tasmania, 17 Liverpool St., Hobart, TAS 7000 Australia; 2 Institute for Physical Activity and Nutrition, Deakin University, Geelong, Australia; 3 Institute of Transport and Logistics Studies, University of Sydney, Sydney, Australia; 4 Metro Tasmania, Hobart, Australia; 5 School of Clinical Sciences, Monash University, Melbourne, Australia; 6 School of Exercise and Nutrition Sciences, Deakin University, Geelong, Australia

**Keywords:** Commuting, Exercise, Public health, Transportation, Translational medical research, Behaviour

## Abstract

**Background:**

Public transport users tend to accumulate more physical activity than non-users; however, whether physical activity is increased by financially incentivising public transport use is unknown. The *trips4health* study aimed to determine the impact of an incentive-based public transport intervention on physical activity.

**Methods:**

A single-blinded randomised control trial of a 16-week incentive-based intervention involved Australian adults who were infrequent bus users (≥ 18 years; used bus ≤ 2 times/week) split equally into intervention and control groups. The intervention group were sent weekly motivational text messages and awarded smartcard bus credit when targets were met. The intervention group and control group received physical activity guidelines. Accelerometer-measured steps/day (primary outcome), self-reported transport-related physical activity (walking and cycling for transport) and total physical activity (min/week and MET-min/week) outcomes were assessed at baseline and follow-up.

**Results:**

Due to the COVID pandemic, the trial was abandoned prior to target sample size achievement and completion of all assessments (*N* = 110). Steps/day declined in both groups, but by less in the intervention group [-557.9 steps (-7.9%) vs.-1018.3 steps/week (-13.8%)]. In the intervention group, transport-related physical activity increased [80.0 min/week (133.3%); 264.0 MET-min/week (133.3%)] while total physical activity levels saw little change [35.0 min/week (5.5%); 25.5 MET-min/week (1.0%)]. Control group transport-related physical activity decreased [-20.0 min/week (-27.6%); -41.3 MET-min/week (-17.3%)], but total physical activity increased [260.0 min/week (54.5%); 734.3 MET-min/week (37.4%)].

**Conclusion:**

This study found evidence that financial incentive-based intervention to increase public transport use is effective in increasing transport-related physical activity These results warrant future examination of physical activity incentives programs in a fully powered study with longer-term follow-up.

**Trial registration:**

This trial was registered with the Australian and New Zealand Clinical Trials Registry August 14th, 2019: ACTRN12619001136190; https://www.anzctr.org.au/Trial/Registration/TrialReview.aspx?id=377914&isReview=true

**Supplementary Information:**

The online version contains supplementary material available at 10.1186/s12966-023-01500-7.

## Background

Physical inactivity is one of the most significant contributing factors of ill-health [1]. In Australia only 24.5% of adults aged 18—64 years met Australian Physical Activity and Sedentary Behaviour Guidelines of 150 min of moderate-to-vigorous physical activity and two or more days containing strength-based training each week [2]. There is an important need for the development of strategies and interventions to increase physical activity levels as Australian physical activity levels have remained stagnant across the last four decades [3].

Relative to leisure-time physical activity, there has been limited investigation of interventions to increase transport-related physical activity [4]. Transport-related physical activity, also known as active transport, consists of healthy means of travel such as walking and cycling (independent or coupled with public or private transport). Due to a need to transit from one place to another (i.e., a necessary and often habitual activity) transport-related physical activity has been identified as a domain in which overall physical activity levels may be increased via the incorporation of physical activity into daily life [5, 6].

The potential contribution of public transport use (such as train/rail, ferry, and bus) towards transport-related physical activity and physical activity is largely realised through additional incidental walking (and potentially cycling in certain contexts) associated with access/egress. In general, people are willing to walk 5–10 min to a stop/station, although this is subject to a range of factors pertaining to the type of service, trip purpose etc. [7]. In our earlier study of Tasmanian bus users, bus passengers self-reported walking 37.8 min per week on average to and from bus stops [8]. Evidently, an increase of even one bus trip per week has the potential for large physical activity gains, at a population level, with each additional public transport trip per week estimated to require 5–15 min of walking to reach a bus stop [8]. As such, transport-related physical activity as a result of public transport use may provide an opportune behavioural target for increasing overall physical activity. Operating through the dual process theory of habituation (i.e., where the conscious decision to use public transport results in the unconscious undertaking of physical activity [9]), public transport promotion provides an opportunity to increase physical activity levels via a habitual association with daily commutes. However, understanding of how public transport use, and consequently transport-related physical activity may be increased, is limited.

One promising method of intervention is the incentivisation of physical activity through financial reward. Previously, financial incentive-based intervention has been successfully applied to alter health behaviours [10], including vaccination [11] and leisure-time physical activity [12, 13]. Grounded in behavioural economics, incentive-based intervention acknowledges the tendency to favour more immediate than long term motivations and instead provides an immediate reward (financial gain) as a key motivator [14]. It is upon these principles, along with input from policy and practice partners, that the *trips4health* study, a single-blinded randomised control trial (RCT) was designed. *trips4health* aimed to determine the impact of a financial incentive-based strategy on transport-related and total physical activity via increased public transport use.

It was hypothesised that intervention group participants would increase steps taken each day and levels of self-reported transport-related physical activity (thus increasing total physical activity), compared to control group participants.

## Methods

### Study design

The* trips4health* study was a single-blinded RCT involving adults undertaking infrequent public transport use (≤ 2 trips per week) in the state capital (greater Hobart region) of Tasmania, Australia [15]. Within this region, public transport was comprised of only bus services, predominantly offered by one provider, Metro Tasmania Pty. Ltd. [16]. Using an incentive-based intervention, the *trips4health* study provided an immediate reward (financial gain) as a motivator for the uptake of public transport usage. The study protocol [15], acceptability and feasibility [16], and positive impact on bus use in the control vs intervention group [17] has been described in detail elsewhere; process evaluation findings indicated that the *trips4health* study demonstrated strong fidelity, feasibility, and acceptability [16].

**Fig. 1:**
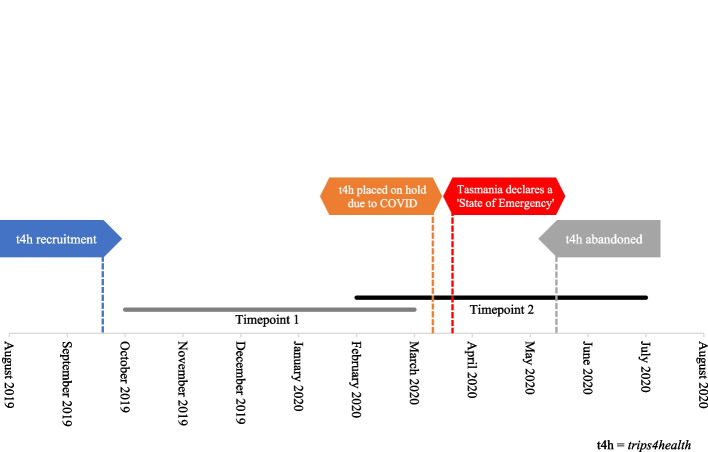
Timeline of trips4health and COVID-19 related changes

Recruitment of participants from the Hobart area began in September 2019 using convenience methods including bus advertising, both social and traditional media promotion, word-of-mouth, workplace promotion, and promotion amongst professional networks. The study was abandoned after approximately one third of the target sample had been recruited due to the COVID-19 pandemic. In March 2020, a state of emergency was announced by the Tasmanian government, and social restrictions enacted (all Tasmanians were asked to stay at home and schools moved to online learning); consequently, the *trips4health* recruitment process was placed on hold in March 2020 (Fig. 1). Follow-up assessments continued in a non-face-to-face format for those that completed the intervention phase prior to the study being paused – this only resulted in alterations to clinical assessments as self-reported participant characteristics and physical activity measures were assessed online electronically at baseline. Due to extensive social changes necessitated by the COVID-19 pandemic, safety concerns, and questions of study validity (participants choosing not to undertake transport-related physical activity due to social changes and/or safety concerns despite entering the intervention phase of the study) investigators chose to abandon the RCT and cease assessments in June 2020 [16].

Ethics approval was granted by the Tasmanian Health and Medical Human Research Ethics Committee (approval number H0017820, 27 March 2019). This trial was registered with the Australian and New Zealand Clinical Trials Registry August 14th, 2019: ACTRN12619001136190; *Universal Trial Number:* U1111-1233–8050. This study was reported according to CONSORT (Consolidated Standards of Reporting Trials) guidelines [18]; CONSORT statement and TIDIER checklists are presented within the supplementary material (Additional file 1, Tables S1 and S2).

### Study participants

Participants were eligible for inclusion in the study upon meeting the following criteria: (i1) ≥ 18 years old; (i2) sufficient English proficiency to provide informed consent; (i3) living in Southern Tasmania; (i4) able to access an urban Tasmania bus service; (i5) must be making trips by motor vehicle that could be made by bus; (i6) current infrequent bus user (on average ≤ 2 trips per week in the past six months); (i7) possession or willingness to possess a public transport smartcard known as Greencard; (i8) willingness for the public transport provider and the researchers to access smartcard data; and (i9) possession of a mobile phone.

Potential participants were excluded if any of the following criteria were met: (e1) intention to move house or work location whereby a bus service in the greater Hobart region will be inaccessible within the 10-month study period; (e2) currently engaged in or planning to engage in other incentive-based programs to enhance public transport use; (e3) pregnancy; (e4) a health condition that prevents walking; (e5) a health condition that prevents bus use; (e6) planned activity that would prevent bus use for greater than two weeks during the four month intervention phase of the 10 month study period (e.g. surgery, extended holiday).

Participant consent was provided electronically through REDCap (Research Electronic Data Capture, Version 8.5.19, Nashville, Tennessee, USA) or in person with research staff when attending the first clinic visit (T1).

### Intervention

The *trips4health* study involved a four-month incentive-based program to increase public transport use between T1 (0 months [baseline]), and T2 (4 months) [15]. The four month intervention phase allowed three months for behaviour change to become ingrained and a further month for maintenance, over which text-message contact and support was reduced and withdrawn in preparation for intervention cessation [19]. A six-month maintenance phase (T3, 10 months) was planned to follow the intervention phase but was not undertaken due to the COVID instigated abandonment of the trial.

All participants (irrespective of group allocation) received smartcard credit as study compensation. Compensation was commensurate with participation and independent of incentive scheme: completion of the baseline assessment (T1) = $5 credit and completion of the post-intervention follow-up assessment (T2) = $10 credit [15].

The intervention used a ‘gain-framed’ approach which rewards positive behaviours. Participants were set weekly bus trip targets from weeks 1–16 and, if intervention group participants met their target number of bus trips, bus trip credit (financial incentive) was issued to their smartcard. The number of target trips increased over time; with this escalation, financial incentives also increased (Additional file 1, Table S3).

Participants were set a target of five one-way bus trips per week by the end of the intervention phase. The credit (incentive) received was equal to the value of the weekly trip target (adjusted to the participant’s usual fare type) [15]. Each week, participants were notified by email whether they met their trip target, the smartcard credit they have received (if any), and their trip target for the following week. To assist in the achievement of weekly targets, and support the retention of public transport-use following the cessation of the intervention [20], additional behaviour change support was delivered to intervention group participants in weekly text messages informing participants of the consequences of transport-related physical activity behaviours, goal setting, and providing social support [21]. These text messages were developed using the Behaviour Change Technique Taxonomy, informed by previous research assessing transport behaviour and physical activity [22] and a prior incentive-based study designed to increase weekly leisure-time physical activity [23].

Both the intervention and control groups received printed versions of the Australian Physical Activity and Sedentary Behaviour Guidelines [5]. Educational materials were to assist in the retention and engagement of both the control and intervention groups but are unlikely to influence physical activity behaviour [24].

### Outcomes

The primary outcome was change in median daily step count. This was assessed via ActiGraph GT3X accelerometers worn on the right hip for seven days at baseline (T1) and immediately post-intervention (week 17/18; T2; primary endpoint). Median daily step count (steps/day) was then derived using ActiLife v6.13.3 analysis software (ActiGraph, Pensacola, FL, USA).

The secondary outcomes of change in self-reported transport-related physical activity and total physical activity (minutes/week and MET-minutes/week) were assessed at baseline and post-intervention via the International Physical Activity Questionnaire -long version (IPAQ-L) [25]. Frequency, intensity, duration, and type of physical activity completed in the previous week was reported, from which self-reported minutes/week and MET-minutes/week of transport-related physical activity undertaken were then calculated according to the IPAQ data processing and analysis protocol [26]. Transport-related physical activity was contextualising within the IPAQ-L as travel “from place to place, including to places like work, stores, movies, and so on.”

Tertiary outcomes of change in self-reported leisure-time physical activity (minutes/week and MET-minutes/week) and sedentary time (min/day) were assessed via IPAQ-L. An objective assessment of sedentary time (min/day) was also determined using ActiGraph GT3X accelerometers.

### Participant characteristics

#### Self-reported measures

Self-reported measures were assessed via an online questionnaire. Age (years) was derived from date of birth and gender was reported as man, woman, trans or other. Highest level of education was self-reported and categorised as: low (year 12 or less); medium (trade/apprenticeship, certificate/diploma); and high (university degree, higher degree). Employment status was categorised as: full time; part time; not employed/working. Number of dependents was reported, while marital status was categorised as: married (living in a registered marriage/a de facto relationship); and not married (separated, divorced, widowed; never married). Self-reported health was categorised into three levels: excellent/very good, good, and fair/poor. Smoking status was self-reported as: current smoking status (yes or no). Access to a private motor vehicle (e.g., car, motorbike) was reported (i.e., yes, yes but some of the time, and no).

#### Clinical measures

Participants’ height was measured to the nearest 0.1 cm via fixed stadiometer (93% MedTec, 7% Charder). Weight was assessed to the nearest 0.1 kg using electronic UC-321PL Precision Scales (94% A & D Medical, 6% Heine). Body mass index was calculated using weight (kg)/height (m)^2^.

#### Smartcard measures

The mean number of bus trips made per week were derived from objective smartcard travel data for 10 weeks prior to randomisation.

### Sample size

Based on the primary outcome of accelerometer-measured steps/day, a target sample size of 300 participants was estimated to provide 80% power with an alpha of 0.05 to detect a difference of 624 steps/day between control and intervention groups; this calculation was based on the mean and standard deviation of steps/day (8000 ± 3500) of data from a sample of participants using the same measure [15, 27]. As such, the *trips4health* study intended to recruit 350 participants (to allow for attrition). Upon study abandonment, 110 participants had completed assessment at T1 and 64 had completed T2. No participants completed the T3 assessment.

### Randomization and blinding

#### Sequence generation

Following completion of the baseline assessment (T1), participants were randomly allocated to the control or intervention group. The allocation sequence was created using computer-generated random numbers on a 1:1 ratio without stratification. Randomisation was conducted in blocks of four.

#### Allocation concealment and implementation

Due to the nature of the intervention, participants could not be blinded to treatment allocation. The details of sequence generation and group allocation were unavailable to research team members bar one unblinded research assistant. The unblinded research team member that created the randomisation sequence had no contact with participants and was not involved with data collection or analysis. The unblinded research assistant initiated the treatment allocation sequence using REDCap and assigned participants to the intervention or control groups.

### Statistical methods

Analysis was performed using STATA version 17.0 (StataCorp LP, College Station, Texas, USA). Participant characteristics of each group were presented as means and standard deviations (SD) for continuous variables and as percentages and frequencies for categorical variables. Physical activity outcomes at baseline and follow-up were presented as medians and interquartile ranges (IQR). Two analyses were performed: all participants that completed T1 baseline physical activity assessment were included within the primary analysis (using multiple imputation), while only those that had also completed the 16-week intervention and T2 follow-up assessments were included within the supplementary complete-case analysis. Missing data at follow-up was addressed via multiple imputation using chained equations using fully conditional specification; assuming missing at random 20 imputations were produced. Analysis of change was then performed to compare differences in average daily step count (primary outcome), total self-reported physical activity and transport-related physical activity levels at follow-up between control and intervention groups. Regression analyses were adjusted for baseline values and undertaken on an intention-to-treat basis. Supplementary complete-case analysis, and analysis of change in self-reported leisure-time physical activity, and objective and subjective measures of sedentary levels are presented in Additional file 1 (Table S4, S5, and S6; Figure S1). Due to this study’s lower than expected sample size, statistical significance could not be confidently ascertained.

## Results

### Participants

**Fig. 2:**
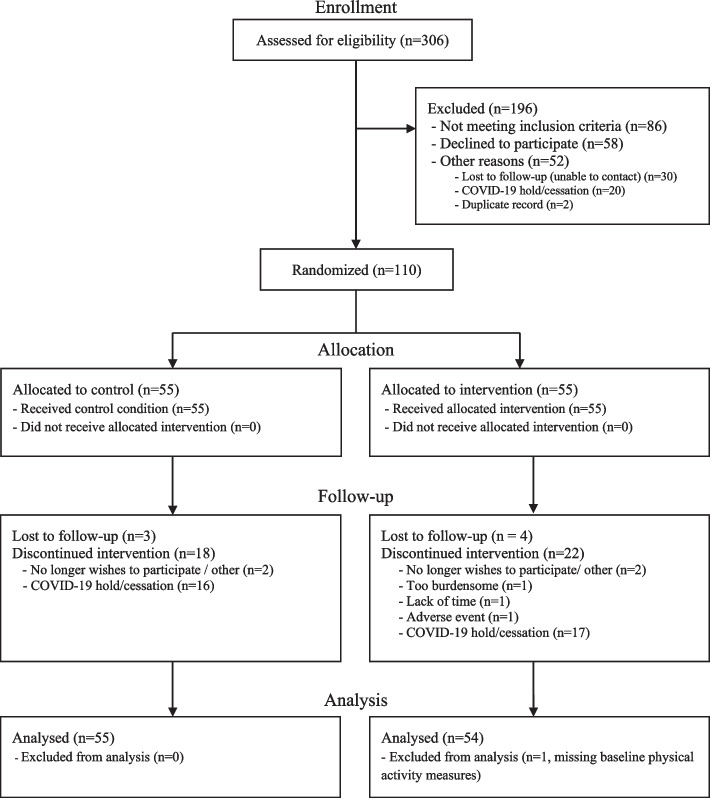
CONSORT Flow Diagram of Participation

The flow of participants through this study is described in Fig. 2. Of those that underwent eligibility screening (N = 306), 110 completed baseline assessment and randomisation. Fifty-five participants (50%) were allocated to the intervention group and 55 to the control group. Steps/day were assessed by accelerometer among 87 participants (41 control and 46 intervention) at baseline and 56 participants (29 control and 27 intervention) at follow-up (immediately post-intervention). Self-report assessments of physical activity and transport-related physical activity were available for 109 participants (54 control and 55 intervention) at baseline and 63 participants (34 control and 29 intervention) participants at follow-up. Results of supplementary complete case analysis were observed to reflect findings presented within this primary analysis (Additional file 1, Table S4).

### Baseline characteristics

Baseline characteristics of control and intervention group participants are presented in Table 1. A greater percentage of control group participants (34.5%) were employed full-time compared to those of the intervention group (18.2%). A lower percentage of participants were married/partnered in the control group (43.6%) than the intervention group (65.4%). All other variables were similar between control and intervention groups participants.

**Table 1:** Participant characteristics at baseline

	Control (*n* = 55)	Intervention (*n* = 55)
Age	44.9 (16.4)	44.2 (17.5)
Sex (male), % (n)	34.5 (19)	27.3 (15)
Body mass index (kg/m^2^)	29.2 (6.7)	27.0 (5.7)
Current smoker, % (n)		
Yes	10.9 (6)	5.5 (3)
No	89.1 (49)	94.5 (52)
Self-rated health		
Excellent/very good	25.9 (14)	41.5 (22)
Good	31.5 (17)	26.4 (14)
Fair/poor	42.6 (23)	32.1 (17)
Highest level of education, % (n)		
High	58.2 (32)	52.7 (29)
Medium	27.3 (15)	21.8 (12)
Low	14.5 (8)	25.4 (14)
Employment status		
Full time	34.5 (19)	18.2 (10)
Part time	38.2 (21)	44.4 (25)
Not employed/working	27.3 (15)	36.4 (20)
Marital status, % (n)		
Married/partnered	43.6 (24)	65.4 (36)
Not married/partnered	56.4 (31)	34.6 (19)
Number of dependents, % (n)	2.0 (1.3)	2.4 (1.6)
Vehicle access, % (n)		
Yes	76.4 (42)	80.0 (44)
Yes, but only some of the time	14.5 (8)	12.7 (7)
No	9.1 (5)	7.3 (4)
Bus trips per week	1.7 (2.0)	1.7 (1.7)

Data presented as mean (SD) unless otherwise indicated

### Outcomes and estimations

Total and transport-related physical activity for each treatment group at baseline and post-intervention follow-up are presented in Table 2.

**Table 2:** Physical activity outcomes at baseline and follow-up, stratified by treatment group

	Control	Intervention
Accelerometer derived steps/day	(*n* = 41)	(*n* = 46)
Pre-intervention	7776.6 (5607, 9742)	7078.6 (6291, 8546)
	(*n* = 29)	(*n* = 27)
Post-intervention	6758.3 (4730, 9197)	6520.7 (5616, 7986)
Transport-related physical activity, minutes/week	(*n* = 54)	(*n* = 55)
Pre-intervention	72.5 (40, 120)	60.0 (30, 210)
	(*n* = 34)	(*n* = 29)
Post-intervention	52.5 (15, 125)	140.0 (40, 240)
Total physical activity, minutes/week	(*n* = 54)	(*n* = 55)
Pre-intervention	477.5 (300, 1151)	635.0 (420, 855)
	(*n* = 34)	(*n* = 29)
Post-intervention	737.5 (400, 1130)	600.0 (470, 895)
Transport-related physical activity, MET-minutes/week	(*n* = 54)	(*n* = 55)
Pre-intervention	239.3 (132, 396)	198.0 (99, 693)
	(*n* = 34)	(*n* = 29)
Post-intervention	198.0 (50, 792)	462.0 (132, 792)
Total physical activity, MET-minutes/week	(*n* = 54)	(*n* = 55)
Pre-intervention	1965.0 (1187, 4611)	2500.0 (1710, 3533)
	(*n* = 34)	(*n* = 29)
Post-intervention	2699.3 (1404, 4110)	2525.5 (1656, 3757)

Data presented as median (inter-quartile range)

Median steps/day decreased from baseline to follow-up in both control (-1018.3 steps; 13.8% reduction) and intervention (-557.9 steps; 7.9% reduction) groups, transport-related physical activity decreased in the control group (20.0 min/week (27.6%)) but increased in the intervention group (80.0 min/week (133.3%)), and total PA increased in the control group (260.0 min/week (54.5%)) and decreased in the intervention group (35.0 min/week (5.5%)) (Table 2).

Analysis of the effect of the incentive intervention on physical activity using imputed data is presented in Table 3; complete case analysis is presented within Additional file 1, Table S4.

**Table 3:** Effect of the incentive intervention on physical activity at follow-up

	n	β (95% CI)
Accelerometer derived mean steps/day	87	-352.1 (-2213.0, 1508.8)
Transport-related physical activity, min/week	109	14.5 (-245.0, 274.1)
Total physical activity, min/week	109	-9.8 (-242.4, 222.7)
Transport-related physical activity, MET-min/week	109	58.0 (-877.0, 992.9)
Total physical activity, MET-min/week	109	-41.9 (-1020.9, 937.2)

β = beta coefficient representing the difference in outcome at follow-up in intervention group vs. control group (reference), adjusted for baseline values. 95% CI = 95% Confidence Interval

#### Accelerometer measured steps/day

Analysis of change observed no statistical significance in steps/day at follow-up between intervention and control groups after adjusting for baseline values: intervention group participants recorded on average 352.1 more steps/day (β = -352.1; 95% Confidence Interval (CI) = -2213.0, 1508.8) than control group participants (Table 3).

#### Self-reported transport-related physical activity

Treatment allocation resulted in a non-significant 14.5 min/week (β = 14.5; 95%CI = -245.0, 274.1) and 58.0 MET-min/week (β = 58.0; 95%CI = -877.0, 992.9) increase in transport-related physical activity among intervention group participants compared to control group participants (Table 3).

#### Self-reported total physical activity

Analysis of change found no significant differences in total physical activity change between intervention and control group participants in either min/week (β = -9.8; 95%CI = -242.4, 222.7) or MET-min/week (β = -41.9; 95%CI = -1020.9, 937.2) (Table 3).

## Discussion

The *trips4health* study is the first RCT to examine the impact of an incentives-based intervention on public transport use for physical activity gain. While a lack of statistical power due to the COVID pandemic makes firm conclusions difficult, contrary to our hypothesis, the median number of steps/day decreased from baseline to follow-up in both treatment groups, but the greatest decrease was observed in the control group. Self-reported transport-related physical activity only increased among the intervention group suggesting some effectiveness of a financial incentive on this physical activity domain; however total physical activity levels decreased among the intervention group while increasing among the control group. Despite the COVID-19 related limitations experienced in this study, these findings for physical activity in combination with our previous observations demonstrating the feasibility of financial incentive to increase bus use [17] warrant further investigation via a fully powered trial.

Despite the established positive relationships between public transport and physical activity [28–30], in the current study accelerometer derived steps/day decreased from baseline to follow-up in both treatment groups. The reason for this decline is unclear but is possibly resultant of a reduction in public transport use (and subsequently the number of steps undertaken to access public transport) due to concerns of safety arising from the escalation of the COVID-19 pandemic [31]. Further, as steps/day was representative of total physical activity, it is possible that this decline may be explained by reductions in occupational and leisure-time activity, driven by COVID-19 work-from-home and stay-home mandates. However, following adjustment for baseline values, a smaller decrease in steps/day was observed among intervention participants compared to those of the control group, suggesting that the financial incentive may have mitigated some declines in physical activity.

Self-reported transport-related physical activity (min/week and MET-min/week) increased among intervention participants by 133.3% while transport-related physical activity of the control group decreased from baseline to follow-up. The differences in self-reported transport-related physical activity (non-significant) between control and intervention group participants may be attributed to the greater number of public transport trips undertaken as a result of the intervention, as commutes between bus stops and destinations constitute transport-related physical activity. In line with the hypothesised dual-process theory mechanism, findings of increased transport-related physical activity performance mirror Cleland et al.’s [17] observations that the financial incentive afforded within the trips4health study yielded an increased number of public transport trips. Due to the target of the intervention (public transport use) transport-related physical activity is conceptually the domain of physical activity in which changes in activity levels would be expected [32].

The self-reported total physical activity levels of the control group increased in both the min/week and MET-min/week undertaken. While a small increase in MET-min/week of total physical activity was reported among the intervention group, a decrease in min/week total physical activity was observed. The intervention group’s increase in transport-related physical activity and relatively unaltered total physical activity suggests the potential for a compensatory mechanism. These patterns are similar to those of the *ActivityStat* hypothesis [33], in which an increase or decrease in one physical activity domain results in a compensatory change in another domain (e.g., transport-related physical activity increases, leisure-time physical activity decreases) thus, resulting in a relatively unchanged total physical activity level. Within this study, transport-related physical activity undertaken by intervention group participants increased by 80.0 min/week, yet the total physical activity of the participants decreased by 35.0 min/week.

While some studies provide evidence of both individual short-term and population-wide secular compensatory mechanisms between physical activity domains [34–37], others are inconclusive [38], suggest no compensation occurs, or observe that increases in one physical activity domain may yield increases in others [35, 39]. These findings add to the already debated field of literature discussing the relationships between physical activity domains.

### Strengths and limitations

This study had some limitations. Importantly, due to the COVID-19 pandemic, the full sample size was not attained, resulting in a lack of statistical power to detect differences between groups. Further, rising concerns regarding COVID-19 during the study period could have impacted on transport and physical activity behaviours. As such, this analysis was unable to draw firm conclusions about the findings, although these data provide some indication of a positive effect and help to inform future studies. COVID-19 also resulted in the abandonment of the planned further post-intervention (6-months) follow-up; thus, it could not be determined whether public transport behaviours would become a more normalised and integrated behaviour over time. The generalisability of this study to the broader population may be limited due to an over-representation of tertiary educated and female participants. The single-blinded design meant that study participants were aware of their group allocation (intervention or control). It must be noted that accelerometer and self-reported physical activity measures differed within this study. For example, it is possible that the control group’s increase in total self-reported physical activity and decrease in ambulatory steps/day is due to activity being undertaken that contributed to total physical activity levels that was not assessable by accelerometer (e.g., cycling, swimming, weightlifting, etc.). The outcomes of transport-related physical activity and total physical activity are prone to limitations due to their self-reported nature. While group allocation had not occurred at T1, participants were aware of their group at the T2 follow-up assessment, which may have biased reported activity levels. However, the use of accelerometers limit potential group allocation bias by providing an objective measure of activity for consideration alongside subjective self-report. There was potential for reactivity bias during the use of accelerometers to measure activity; However, the assessment of activity across a seven-day period and the analysis of the median steps/day over this period act to negate this potential bias. Further, as accelerometers were mailed to participants at T2 rather that administered face-to-face (as at T1), compliance with instruction of use may have been lower.

A key strength of this study was its randomised and controlled study design. Further, its use of both objective and subjective measures of physical activity were important strengths. Accelerometers are an accurate and reliable objective measure of both step count and physical activity frequency, intensity, and duration [40, 41]. This study also assessed physical activity subjectively using the IPAQ-L, a widely implemented survey that has been shown to be both valid and reliable [25, 42], which allowed for the separation of transport-related and leisure-time physical activity [25]. The use of multiple imputation strengthened the analysis by allowing for the consideration of missing outcome data.

## Conclusions

Our findings suggest that this financial incentive-based intervention to increase public transport use and subsequently physical activity has demonstrated some evidence of effectiveness in increasing transport-related physical activity. Future examination of the effect of incentives programs on physical activity outcomes in a fully powered study with broader participant reach and a longer examination of behaviour change is warranted.

### Supplementary Information

**Additional file 1: Table S1.** CONSORT 2010 checklist of information to include when reporting a randomised trial. **Table S2. **The TIDieR (Template for Intervention Description and Replication) Checklist*. **Table S3.** Trips4health intervention schedule. **Table S4.** Effect of the incentive intervention on physical activity at follow-up (complete case). **Table S5.** Tertiary activity and sedentary outcomes at baseline and follow-up, stratified by treatment group. **Table S6.** Effect of the incentive intervention on leisure-time physical activity and sedentary behaviour at follow-up. **Figure S1. **Median activity levels stratified by group and timepoint. 

## Data Availability

The datasets used and/or analysed during the current study are available from the corresponding author on reasonable request.

## Abbreviations

CI: Confidence interval
CONSORT: Consolidated Standards of Reporting Trials
COVID19: Coronavirus disease
IPAQ: International physical activity questionnaire
RCT: Randomised control trial
SD: Standard deviation
