# Supplementary material for: trips4health: a single-blinded randomised controlled trial incentivising adult public transport use for physical activity gain
Source: Int J Behav Nutr Phys Act. 2023 Aug 16;20:98. doi: 10.1186/s12966-023-01500-7 (PMC10428598; doi:10.1186/s12966-023-01500-7)
Supplement: Supplementary file 1 — Additional file 1: Table S1. CONSORT 2010 checklist of information to include when reporting a randomised trial. Table S2. The TIDieR (Template for Intervention Description and Replication) Checklist*. Table S3. Trips4health intervention schedule. Table S4. Effect of the incentive intervention on physical activity at follow-up (complete case). Table S5. Tertiary activity and sedentary outcomes at baseline and follow-up, stratified by treatment group. Table S6. Effect of the incentive intervention on leisure-time physical activity and sedentary behaviour at follow-up. Figure S1. Median activity levels stratified by group and timepoint. [file 12966_2023_1500_MOESM1_ESM.doc]

*Trips4health:* Public Transport Use for Physical Activity Gain

Additional File 1


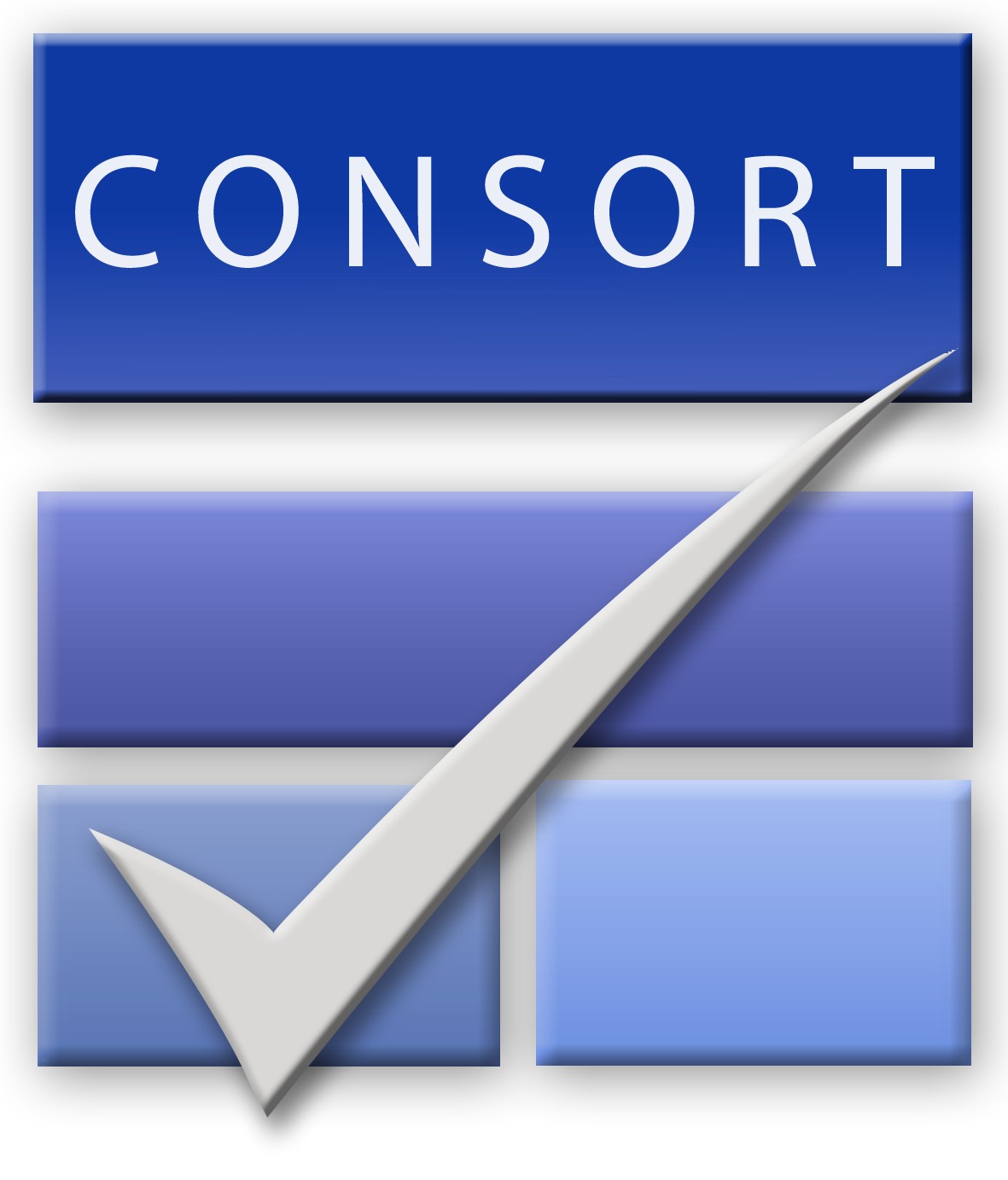


| Table S1. CONSORT 2010 checklist of information to include when reporting a randomised trial | | | |
| --- | --- | --- | --- |
| Section/Topic | Item No | Checklist item | Reported on page No |
| Title and abstract | | | |
|  | 1a | Identification as a randomised trial in the title | 1 |
|  | 1b | Structured summary of trial design, methods, results, and conclusions (for specific guidance see CONSORT for abstracts) | 1 |
| Introduction | | | |
| Background and objectives | 2a | Scientific background and explanation of rationale | 2-3 |
|  | 2b | Specific objectives or hypotheses | 3 |
| Methods | | | |
| Trial design | 3a | Description of trial design (such as parallel, factorial) including allocation ratio | 4-5 |
|  | 3b | Important changes to methods after trial commencement (such as eligibility criteria), with reasons | 5 |
| Participants | 4a | Eligibility criteria for participants | 3-4 |
|  | 4b | Settings and locations where the data were collected | 3-4 |
| Interventions | 5 | The interventions for each group with sufficient details to allow replication, including how and when they were actually administered | 4-6 |
| Outcomes | 6a | Completely defined pre-specified primary and secondary outcome measures, including how and when they were assessed | 6-7 |
|  | 6b | Any changes to trial outcomes after the trial commenced, with reasons | 5 |
| Sample size | 7a | How sample size was determined | 7-8 |
|  | 7b | When applicable, explanation of any interim analyses and stopping guidelines | NA |
| Randomisation: |  |  |  |
| Sequence generation | 8a | Method used to generate the random allocation sequence | 8 |
|  | 8b | Type of randomisation; details of any restriction (such as blocking and block size) | 8 |
| Allocation concealment mechanism | 9 | Mechanism used to implement the random allocation sequence (such as sequentially numbered containers), describing any steps taken to conceal the sequence until interventions were assigned | 8 |
| Implementation | 10 | Who generated the random allocation sequence, who enrolled participants, and who assigned participants to interventions | 8 |
| Blinding | 11a | If done, who was blinded after assignment to interventions (for example, participants, care providers, those assessing outcomes) and how | 8 |
|  | 11b | If relevant, description of the similarity of interventions |  |
| Statistical methods | 12a | Statistical methods used to compare groups for primary and secondary outcomes | 8 |
|  | 12b | Methods for additional analyses, such as subgroup analyses and adjusted analyses | 8 |
| Results | | | |
| Participant flow (a diagram is strongly recommended) | 13a | For each group, the numbers of participants who were randomly assigned, received intended treatment, and were analysed for the primary outcome | 9, Fig 1 |
|  | 13b | For each group, losses and exclusions after randomisation, together with reasons | Fig 1. |
| Recruitment | 14a | Dates defining the periods of recruitment and follow-up | 5, Fig 1 |
|  | 14b | Why the trial ended or was stopped | 5 |
| Baseline data | 15 | A table showing baseline demographic and clinical characteristics for each group | Fig 2 |
| Numbers analysed | 16 | For each group, number of participants (denominator) included in each analysis and whether the analysis was by original assigned groups | 9, Table 2, Table 3 |
| Outcomes and estimation | 17a | For each primary and secondary outcome, results for each group, and the estimated effect size and its precision (such as 95% confidence interval) | 13 |
|  | 17b | For binary outcomes, presentation of both absolute and relative effect sizes is recommended | NA |
| Ancillary analyses | 18 | Results of any other analyses performed, including subgroup analyses and adjusted analyses, distinguishing pre-specified from exploratory | Table S3 |
| Harms | 19 | All important harms or unintended effects in each group (for specific guidance see CONSORT for harms) | NA |
| Discussion | | | |
| Limitations | 20 | Trial limitations, addressing sources of potential bias, imprecision, and, if relevant, multiplicity of analyses | 20 |
| Generalisability | 21 | Generalisability (external validity, applicability) of the trial findings | 17-19 |
| Interpretation | 22 | Interpretation consistent with results, balancing benefits and harms, and considering other relevant evidence | 17-19 |
| Other information | | |  |
| Registration | 23 | Registration number and name of trial registry | 1, 4 |
| Protocol | 24 | Where the full trial protocol can be accessed, if available |  |
| Funding | 25 | Sources of funding and other support (such as supply of drugs), role of funders | 21-22 |

*We strongly recommend reading this statement in conjunction with the CONSORT 2010 Explanation and Elaboration for important clarifications on all the items. If relevant, we also recommend reading CONSORT extensions for cluster randomised trials, non-inferiority and equivalence trials, non-pharmacological treatments, herbal interventions, and pragmatic trials. Additional extensions are forthcoming: for those and for up to date references relevant to this checklist, see

**
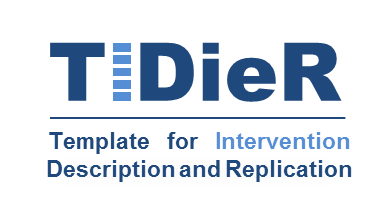
**

| **Table S2. The TIDieR (Template for Intervention Description and Replication) Checklist*:** | |  | |
| --- | --- | --- | --- |
| **Item number** | **Item** | **Where located **** | |
|  |  | Primary paper  (page or appendix  number) | Other ^†^  (details) |
|  | **BRIEF NAME** |  |  |
| **1.** | Provide the name or a phrase that describes the intervention. | Title, Abstract and Page 1 | ______________ |
|  | **WHY** |  |  |
| **2.** | Describe any rationale, theory, or goal of the elements essential to the intervention. | Pages 1-2 | _____________ |
|  | **WHAT** |  |  |
| **3.** | Materials: Describe any physical or informational materials used in the intervention, including those provided to participants or used in intervention delivery or in training of intervention providers. Provide information on where the materials can be accessed (e.g. online appendix, URL). | Pages 3-5 | _____________ |
| **4.** | Procedures: Describe each of the procedures, activities, and/or processes used in the intervention, including any enabling or support activities. | Pages 5-6 | _____________ |
|  | **WHO PROVIDED** |  |  |
| **5.** | For each category of intervention provider (e.g. psychologist, nursing assistant), describe their expertise, background and any specific training given. | N/A | _____________ |
|  | **HOW** |  |  |
| **6.** | Describe the modes of delivery (e.g. face-to-face or by some other mechanism, such as internet or telephone) of the intervention and whether it was provided individually or in a group. | Pages 5-6 | _____________ |
|  | **WHERE** |  |  |
| **7.** | Describe the type(s) of location(s) where the intervention occurred, including any necessary infrastructure or relevant features. | Page 3 and 6 | _____________ |

|  | **WHEN and HOW MUCH** |  |  |
| --- | --- | --- | --- |
| **8.** | Describe the number of times the intervention was delivered and over what period of time including the number of sessions, their schedule, and their duration, intensity or dose. | Pages 5-6  Table S3 | _____________ |
|  | **TAILORING** |  |  |
| **9.** | If the intervention was planned to be personalised, titrated or adapted, then describe what, why, when, and how. | N/A | _____________ |
|  | **MODIFICATIONS** |  |  |
| **10.^ǂ^** | If the intervention was modified during the course of the study, describe the changes (what, why, when, and how). | N/A | _____________ |
|  | **HOW WELL** |  |  |
| **11.** | Planned: If intervention adherence or fidelity was assessed, describe how and by whom, and if any strategies were used to maintain or improve fidelity, describe them. | Page 6,7 | _____________ |
| **12.^ǂ^** | Actual: If intervention adherence or fidelity was assessed, describe the extent to which the intervention was delivered as planned. | doi: [10.1186/s12966-022-01394-x](https://doi.org/10.1186%2Fs12966-022-01394-x)). | _____________ |

** **Authors** - use N/A if an item is not applicable for the intervention being described. **Reviewers** – use ‘?’ if information about the element is not reported/not sufficiently reported.

† If the information is not provided in the primary paper, give details of where this information is available. This may include locations such as a published protocol or other published papers (provide citation details) or a website (provide the URL).

ǂ If completing the TIDieR checklist for a protocol, these items are not relevant to the protocol and cannot be described until the study is complete.

* We strongly recommend using this checklist in conjunction with the TIDieR guide (see *BMJ* 2014;348:g1687) which contains an explanation and elaboration for each item.

* The focus of TIDieR is on reporting details of the intervention elements (and where relevant, comparison elements) of a study. Other elements and methodological features of studies are covered by other reporting statements and checklists and have not been duplicated as part of the TIDieR checklist. When a **randomised trial** is being reported, the TIDieR checklist should be used in conjunction with the CONSORT statement (see [www.consort-statement.org](http://www.consort-statement.org/)) as an extension of **Item 5 of the CONSORT 2010 Statement.** When a **clinical trial** **protocol** is being reported, the TIDieR checklist should be used in conjunction with the SPIRIT statement as an extension of **Item 11 of the SPIRIT 2013 Statement** (see [www.spirit-statement.org](http://www.spirit-statement.org/)). For alternate study designs, TIDieR can be used in conjunction with the appropriate checklist for that study design (see [www.equator-network.org](http://www.equator-network.org/)).

| Table S3. Trips4health intervention schedule | | | | | |
| --- | --- | --- | --- | --- | --- |
|  | Intervention phase | Bus trip targets | Smartcard credit available (adult all zones fare^a^)  AUD$ | Smartcard credit available (concession all zones fare^b^) | Text message frequency |
| Within 2 weeks  pre-intervention | *Baseline survey, clinic assessment, accelerometer* | | | | |
| Week 1 | Getting started | 1 | $5.76 | $1.92 | x 2 |
| Week 2 | Getting started | 1 | $5.76 | $1.92 | x 2 |
| Week 3 | Slowly increasing | 2 | $11.52 | $3.84 | x 2 |
| Week 4 | Slowly increasing | 2 | $11.52 | $3.84 | x 2 |
| Week 5 | Moving along | 3 | $17.28 | $5.76 | x 2 |
| Week 6 | Moving along | 3 | $17.28 | $5.76 | x 2 |
| Week 7 | Moving along | 3 | $17.28 | $5.76 | x 2 |
| Week 8 | Moving along | 3 | $17.28 | $5.76 | x 2 |
| Week 9 | Aiming high | 4 | $23.04 | $7.68 | x 2 |
| Week 10 | Aiming high | 4 | $23.04 | $7.68 | x 2 |
| Week 11 | Aiming high | 4 | $23.04 | $7.68 | x 2 |
| Week 12 | Aiming high | 4 | $23.04 | $9.60 | x 2 |
| Week 13 | Keeping up | 5 | $28.80 | $9.60 | x 1 |
| Week 14 | Keeping up | 5 | $28.80 | $9.60 | x 1 |
| Week 15 | Keeping up | 5 | $28.80 | $9.60 | x 1 |
| Week 16 | Keeping up | 5 | $28.80 | $9.60 | x 1 |
| ***Total*** | | **55** | **$316.80** | **$105.60** | **28** |
| Week 17 or 18 | *Post-intervention survey, clinic assessment, accelerometer,* | | | | |
| Week 41 or 42  (10 months from baseline) | *Follow-up survey, clinic assessment, accelerometer* | | | | |

| Table S4. Effect of the incentive intervention on physical activity at follow-up (complete case) | | |
| --- | --- | --- |
|  | n | β (95% CI) |
| Accelerometer derived mean steps/day | 50 | -334.9 (-1843.7, 1172.9) |
| Transport-related physical activity, min/week | 63 | 24.5 (-98.4, 147.3) |
| Total physical activity, min/week | 63 | 16.7 (-206.8, 239.2) |
| Transport-related physical activity, MET-min/week | 63 | 81.4 (-355.3, 518.2) |
| Total physical activity, MET-min/week | 63 | 120.3 (-832.8, 1073.3) |
| β = beta coefficient representing the difference in outcome at follow-up in intervention group vs. control group, adjusted for baseline values  95 % CI = 95 % Confidence Interval | | |

| Table S5. Tertiary activity and sedentary outcomes at baseline and follow-up, stratified by treatment group | | |
| --- | --- | --- |
|  | Control | Intervention |
| Leisure-time physical activity, min/week | (n=54) | (n=55) |
| Pre-intervention | 90.0 (20, 270) | 180.0 (40, 300) |
|  | (n=34) | (n=29) |
| Post-intervention | 80.0 (0, 291) | 135.0 (90, 210) |
|  |  |  |
| Leisure-time physical activity,  MET-minutes/week | (n=54) | (n=55) |
| Pre-intervention | 313.5 (66, 1294) | 693.0 (132, 1848) |
|  | (n=34) | (n=29) |
| Post-intervention | 301.5(0, 1188) | 594 (297, 1080) |
|  |  |  |
| Self-reported sitting time excluding during transport (min/week) | (n=54) | (n=55) |
| Pre-intervention | 2880 (2160, 4260) | 2280 (1470, 3120) |
|  | (n=34) | (n=29) |
| Post-intervention | 2655 (1800, 3600) | 2100 (1680, 3120) |
| Self-reported total sitting time including during transport (min/week) | (n=54) | (n=55) |
| Pre-intervention | 3150 (2610, 4519) | 2560 (1720, 3360) |
|  | (n=34) | (n=29) |
| Post-intervention | 3035 (2380, 4040) | 2640 (1730, 3720) |
|  |  |  |
| Accelerometer-measured total sedentary time (min/day) | (n=41) | (n=46) |
| Pre-intervention | 544.6 (427, 612) | 504.2 (465, 570) |
|  | (n=29) | (n=27) |
| Post-intervention | 519.3 (468, 598) | 487.1 (377, 578) |
|  |  |  |
| Data presented as median (inter-quartile range) | | |

| Table S6. Effect of the incentive intervention on leisure-time physical activity and sedentary behaviour at follow-up | | |
| --- | --- | --- |
|  | n | β (95% CI) |
| Leisure-time physical activity, min/week | 109 | -2.3 (-119.9, 116.8) |
| Leisure-time physical activity,  MET-minutes/week | 109 | -42.1 (-1222.9, 1158.8) |
| Self-reported total sitting time excluding during transport (min/week) | 109 | 100.0 (-449.0, 649.1) |
| Self-reported total sitting time including during transport (min/week) | 109 | 149.5 (-706.7, 1005.8) |
| Accelerometer-measured total sedentary time (min/day) | 87 | 13.4 (-81.1, 54.2) |
| β = beta coefficient representing the difference in outcome at follow-up in intervention group vs. control group with multiple imputation, adjusted for baseline values  95 % CI = 95 % Confidence Interval | | |


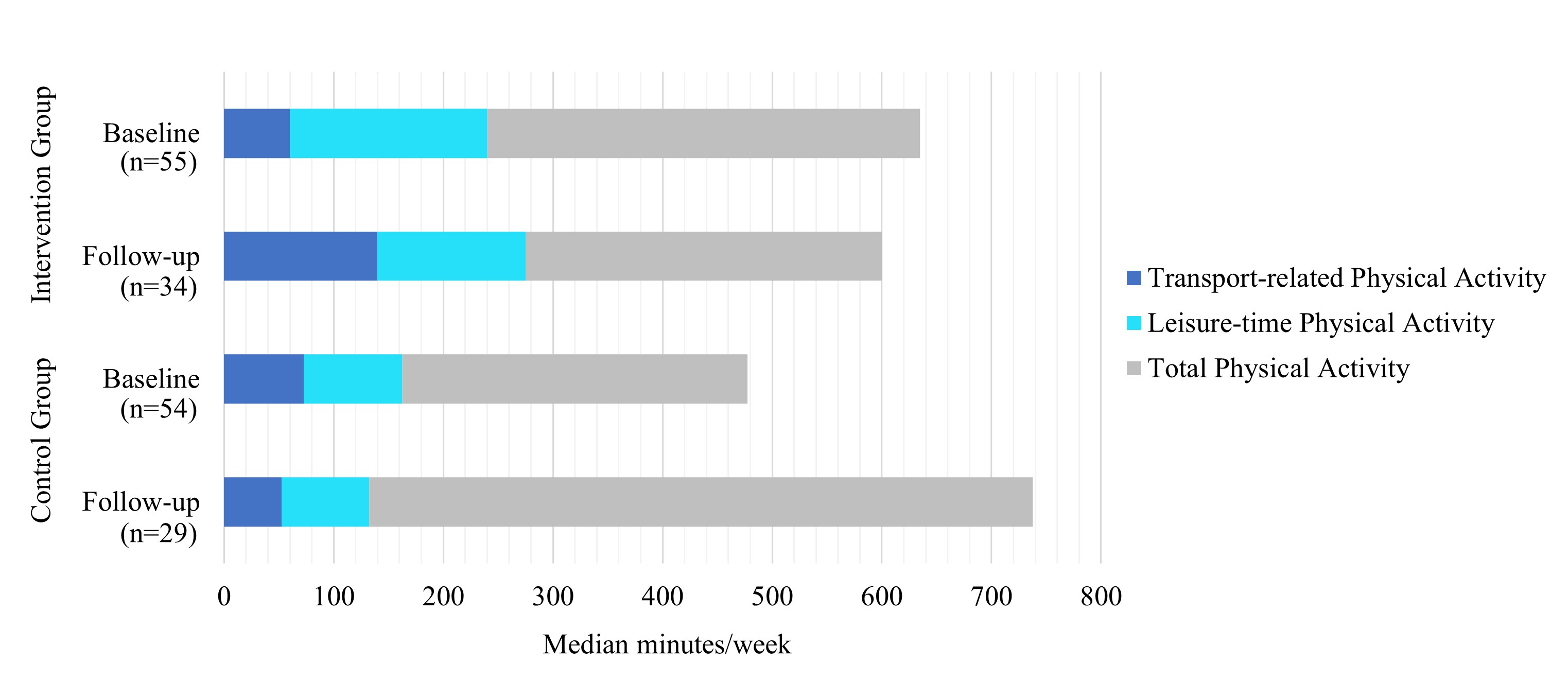
Figure S1. Median activity levels stratified by group and timepoint
